# Quantifying the Contribution of Nondialytic Factors Affecting Predialysis Serum Phosphate Level When Comparing Hemodiafiltration with Hemodialysis

**DOI:** 10.3390/toxins18040179

**Published:** 2026-04-08

**Authors:** John T. Daugirdas

**Affiliations:** College of Medicine, University of Illinois at Chicago, 820 S. Wood St., Chicago, IL 60612, USA; jtdaugir@uic.edu

**Keywords:** hemodiafiltration, phosphate, phosphorus, hyperphosphatemia, kinetic modeling, phosphate binders, phosphate intake, dietary phosphate, phosphate food additives, dialyzer clearance

## Abstract

Hyperphosphatemia is a major complication in patients with kidney failure undergoing dialysis and is strongly associated with cardiovascular disease, vascular calcification, and increased mortality. Conventional management relies on dietary phosphate restriction, oral phosphate binders, and dialysis, yet persistent hyperphosphatemia affects a substantial proportion of patients. High-volume hemodiafiltration, combining diffusive and convective clearances, achieves greater phosphate removal than standard hemodialysis, with kinetic modeling predicting ~15–20% higher dialytic phosphate clearance (and ~0.5 mg/dL lower predialysis serum phosphate when nondialytic factors are constant). In this narrative review, we quantify the magnitude of improvement in dialytic clearance of phosphate with hemodiafiltration relative to hemodialysis and evaluate its effects on phosphate control measures. We also analyze phosphate balance in selected hemodiafiltration vs. hemodialysis comparisons and demonstrate why predialysis serum phosphate levels are sometimes only modestly lower or similar when hemodiafiltration is compared with hemodialysis. These findings are largely attributable to nondialytic factors—minor differences in phosphate binder equivalent dose, dietary phosphate ingestion, or residual kidney function—as predicted by phosphate kinetic modeling and supported by clinical trial data. Recognizing these confounders is essential for interpreting hemodiafiltration’s phosphate-lowering potential in real-world practice.

## 1. Introduction

The burden of chronic kidney disease–mineral and bone disorder (CKD-MBD) in ESKD patients undergoing maintenance dialysis is substantial, with hyperphosphatemia serving as a pivotal driver of adverse outcomes. Defined as predialysis serum phosphate exceeding 5.5 mg/dL (1.78 mmol/L) [1], hyperphosphatemia is prevalent in approximately 50–70% of dialysis patients worldwide [2,3] and is independently associated with mortality (with risk increasing ~20–30% per 1 mg/dL increment in large cohorts) [2,3,4,5], cardiovascular events [6,7], and accelerated vascular calcification [8,9,10]. Low serum phosphate, found in patients with low body mass index, sarcopenia, and low serum albumin levels, is a marker of illness and undernutrition and is also associated with increased mortality risk. Recent data from the Dialysis Outcomes and Practice Patterns Study (DOPPS) indicate that mean predialysis phosphate levels among hemodialysis patients in the United States rose to 5.6 mg/dL by 2021 (from a nadir of ~5.0 mg/dL around 2013) [11]. Perhaps this was related to updated KDIGO guidelines that suggested lowering elevated phosphate only “toward the normal range” [12], while KDOQI previously advocated a target range of 3.5–5.5 mg/dL [1].

Typical intake of phosphate (800–1500 mg/day from foods and additives) in dialysis patients far outpaces dialytic removal, with thrice-weekly hemodialysis extracting only 800–1000 mg per session via primarily diffusive transport. Phosphate, a charged trivalent anion (MW 95 Da), is mobilized slowly from a large intracellular non-plasma compartment during dialysis, resulting in a steep initial intradialytic decline in serum phosphate, followed by a plateau phase that persists even during extended-hour treatments. After dialysis is stopped, a large postdialysis rebound begins almost immediately, perpetuating hyperphosphatemia during the interdialysis period [13].

## 2. Kinetic Models of Phosphate Removal During Extracorporeal Treatment

A large number of kinetic models of phosphate removal during hemodialysis have been proposed, as reviewed in [14,15]. One model developed by Daugirdas (the Solute Solver phosphate model) was partially validated by data obtained during dialysis treatments given during the Hemodialysis (HEMO) study, in which additional laboratory measurements were made in blood drawn during and shortly after dialysis [16]. In that two-pool model, a large distal compartment was postulated, which was necessary to explain the relative plateau phase of serum phosphate observed during the latter part of a dialysis session as well as the large postdialysis rebound. One unique feature of the model was a variable postulated intercompartmental clearance between the large distal compartment and the proximal compartment [16]. As long as the intradialytic serum water phosphate level remained above 3.0 mg/dL, the rate of transfer from the distal to the proximal compartment was modeled to be quite low, but whenever the intradialytic serum water level fell below 3.0, the model postulated that the intercompartmental clearance from the large distal reservoir would increase markedly. With these modifications, the Solute Solver phosphate model explained both the intradialytic and early postdialysis serum phosphate levels that were observed in HEMO study patients [16]. The model was further validated by comparing the mean modeled phosphate removal during dialysis sessions with reported measured mean values in a series of studies published by others [17].

## 3. Removal of Phosphate by Hemodialysis vs. Hemodiafiltration

The dialyzer clearance of phosphate is considerably lower than that for urea. In the Solute Solver phosphate model, the in vitro mass transfer area coefficient of phosphate (K_0_A) is assumed to be 40–60% that of urea. This is because of the slower diffusion of phosphate across dialyzer membranes compared with urea. In older studies, this ratio of in vitro dialyzer K_0_A (phosphate-to-urea) value was assumed to be 0.40, while in more recent studies, it was assumed to be 0.50. In the most recent studies using highly efficient dialyzers in which phosphate clearance has been optimized, an in vitro phosphate-to-urea K_0_A ratio of 0.60 can be assumed [18].

During passage of blood through a dialyzer, phosphate is removed only from the plasma compartment, while urea is removed from both plasma and red blood cells. This further reduces in vivo dialyzer phosphate clearance relative to urea, and this blood compartment-related reduction is magnified when the hematocrit of blood going through the dialyzer is high, reducing the net plasma flow rate [13,18].

The intradialytic plateau phase of serum phosphate increases the benefit of extending dialysis time on phosphate removal. As a result, one of the most useful dialytic methods of increasing phosphate removal is to increase dialysis session length [13].

In treatments using hemodiafiltration, the convective component will increase both urea and phosphate clearances. In the Solute Solver phosphate kinetic model, the convective component of phosphate clearance is calculated using equations for hemodiafiltration clearance proposed by the EuDial group [19].

## 4. Nondialytic Factors Affecting Predialysis Serum Phosphate

In order to understand the effect of hemodiafiltration on the predialysis serum phosphate, it is useful to review several nondialysis factors that affect this value in patients, regardless of the type of dialysis treatment being given.

### 4.1. Dietary Phosphate Ingestion

In urea modeling, the predialysis serum urea nitrogen levels depend on the balance of generation and removal, with urea generation coming from the metabolism of proteins and other nitrogen-containing compounds. With phosphate, the situation is analogous, except that there is no phosphate “generation” per se, but rather, phosphate absorption from dietary intake. In US persons without kidney disease, according to NHANES data [20] from 2015–2016, the average phosphate ingestion was 1400 mg/day (17.5 mg/kg body weight) and was substantially higher in men, at 1600 mg/day (18.7 mg/kg), than in women, at 1190 mg/day (16.2 mg/kg). Phosphate intake is markedly lower in the elderly. In another analysis of NHANES data, in which average adjusted daily phosphate ingestion for all age groups was 1380 mg/day, intake in subjects aged 20–50 years was 1460 mg/day, while in subjects aged more than 70 years, intake was 25% lower, averaging only 1120 mg/day [21].

**Figure 1:**
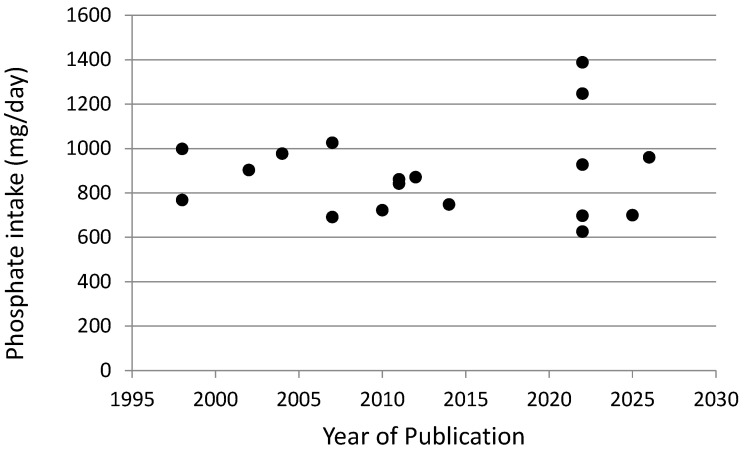
Average daily phosphate ingestion in hemodialysis patients, as reported in 18 studies published between 1995 and 2026. See references [22,23,24,25,26,27,28,29,30,31,32,33,34,35,36,37,38,39]. Mean intake from Noori et al. [28] was calculated by extracting and averaging data from their published phosphate ingestion scatterplot. The daily phosphate intake recorded by the large Diet-HD study of 1388 mg/day by Su et al. [33] is a median value.

With regard to average phosphate ingestion in dialysis patients, reported results [22,23,24,25,26,27,28,29,30,31,32,33,34,35,36,37,38,39] vary, as shown in Figure 1. There seems to have been no major increase reported in mean phosphate ingestion over the 1995–2020 period. A very high median intake value of 1388 mg/day was reported in 2022 by Su et al. [33] in a large (*n* = 8110 patients) European cooperative study, which is higher compared with most other reports. Three publications in which average phosphate ingestion was measured to be less than 700 mg/day were all from Asia; this may have been related to the relatively low body weights of the patients studied and/or the relatively low animal protein and dairy intake in the typical Asian diet. The unweighted average of daily phosphate ingestion in the 18 studies shown in Figure 1 was 900 mg/day, considerably less than the 1400 mg/day average intake reported by NHANES in normal subjects [21], but perhaps not so much less than the 1120 mg/day intake per day reported in subjects without marked kidney disease who were greater than 70 years of age [21], as discussed above.

The main reason that phosphate ingestion is higher in men than in women seems to be related to the greater intake of dietary protein by men. Although the amino acids found in proteins contain no phosphate per se, dietary phosphate ingestion is highly correlated with dietary protein intake [28,37]. This is because dairy, meat, fish, eggs, and poultry contain phosphoproteins, phospholipids (cell membranes), nucleotides/RNA/DNA, ATP, and other phosphate-containing molecules. With plant proteins, the amount of phosphate per mg protein is less than in animal proteins. Also, many plant foods, esp. grains, legumes, and beans, contain phytate, which binds to phosphate, lessening its absorption.

An additional important source of phosphate in the diet is the ingestion of foods admixed with phosphate-containing additives. These compounds are added by food processors to help modify the moisture content, storage freshness, and other features of foods. Currently, the amounts of phosphate additives contained in processed foods are not listed on food labels, making precise assessment of phosphate ingestion by dietary surveys difficult. In one of the NHANES studies of phosphate ingestion discussed above, 12% of ingested phosphate was recorded to have come from food additives [20].

Another source of phosphate to be considered is medications. Phosphate can be present in a tablet or capsule as an active ingredient, counter-ion, or most commonly, excipient (e.g., anhydrous dibasic calcium phosphate, calcium hydrogen phosphate, disodium hydrogen phosphate, or calcium glycerophosphate). These excipients are used as fillers, binders, disintegrants, and buffers. Drug excipient phosphate content is usually unlabeled and varies by manufacturer. Phosphate content may be different in brand-name vs. generic drugs and may vary with different formulations of the same drug. Across various studies, ~11–19% of commonly prescribed drugs have been reported to contain absorbable phosphate [40,41].

### 4.2. Phosphate Absorption

Apart from phosphate ingestion, the assumed percent of ingested phosphate that is absorbed is of key importance to a phosphate kinetic model. In the Solute Solver phosphate model, the assumed percentage of phosphate absorption is fixed at 67%, although this value can be changed. A review of the literature supports an overall estimate of 60–70% phosphate absorption in patients eating a mixed diet and not taking drugs that interfere with phosphate absorption [42]. Treatment with activated vitamin D compounds (e.g., calcitriol) increases gut phosphate absorption. The composition of the diet is important to know because, as mentioned above, the percent phosphate absorbed from plant-based diets is lower than from diets comprised largely of animal proteins, and particularly, those diets that are rich in dairy products. Complicating the quantification of absorbed phosphate is the fact that the percentage of phosphate absorbed from food additives or medications may be quite high.

## 5. Phosphate Binders and the Phosphate Binder Equivalent Dose (PBED)

A substantial minority of hemodialysis patients can maintain predialysis serum phosphate levels in the range associated with the lowest mortality (3.5–5.5 mg/dL) just by limiting ingestion of foods that contain large amounts of phosphate. Many other patients require treatment with a phosphate-binding drug, and some require the simultaneous use of phosphate binders of two different classes. There are six classes of phosphate binders in current use, some containing calcium, and others not. In order to help systematize the evaluation of phosphate binder usage in the Frequent Hemodialysis Network study, the dose of each class of binder estimated to bind 45 mg of phosphate was calculated and named the “phosphate binder equivalent dose” or PBED [43,44]. Because 1 g of calcium carbonate has been found to bind this amount of phosphate, the PBED can also be thought of as the dose of binder that is equivalent to the same number of grams of calcium carbonate. The proposed utility of the PBED concept is that it may aid in dosing phosphate binders when there is a need to switch from one class of binder to another. It may also help compare equivalent doses of phosphate binders in patients who are taking more than one class of binders simultaneously. Typically, in hemodialysis patients, doses of phosphate binders in the 3–6 g/day range are commonly seen [44], although higher doses may be needed in patients in whom phosphate ingestion is high, while lower doses are sufficient in patients with substantial residual kidney function.

## 6. Residual Kidney Phosphate Clearance

Phosphate control is improved in patients with substantial residual kidney function [44,45,46,47]. The residual kidney phosphate clearance is slightly higher (by about 20%) than residual kidney urea clearance, and substantially lower than residual kidney creatinine clearance [48]. In patients with an average predialysis serum phosphate level of 4.5 mg/dL, the amount of phosphate removed in the urine averaged 60 mg/day for each mL/min residual kidney phosphate (water) clearance. An amount of 45 mg of phosphate is the amount bound by a PBED of 1 g. Hence, one can expect that each mL/min of residual kidney phosphate (water) clearance will obviate the need for about 1.3 g/day of phosphate binders (when dose is expressed as PBED), though the amount of reduction will vary somewhat depending on baseline values of renal function and PBED.

## 7. Other Factors Associated with Serum Phosphate

Serum phosphate in dialysis patients is also associated with body mass index, mineral bone disorder, specifically serum parathyroid hormone levels, and other metabolic factors. A review of these is beyond the scope of the present manuscript.

## 8. Do More Efficient Extracorporeal Treatments Remove More Phosphate?

One additional point that may not be obvious to those not familiar with kinetic modeling is that whenever one has a therapy such as hemodiafiltration that is able to clear phosphate more efficiently than hemodialysis, it is tempting to postulate that the enhanced therapy “removes more phosphate.” However, in addition to dialyzer phosphate clearance, another very important determinant of the amount of phosphate removed during an extracorporeal treatment session is the time-averaged intradialytic serum level of phosphate during that treatment, and this, in turn, is closely correlated with the predialysis serum phosphate value. In a head-to-head comparison of hemodiafiltration with hemodialysis, the former will indeed remove more phosphate if the treatments being compared were measured when predialysis serum phosphate values were similar. Once a patient continues on hemodiafiltration, however, the predialysis serum phosphate levels will drop due to this enhanced clearance. Ultimately, a new, lower equilibrium value of predialysis serum phosphate will be established, at which point removal of phosphate by hemodiafiltration and hemodialysis will again be comparable, except for the fact that with hemodiafiltration, the same amount of phosphate is being removed at a lower predialysis and time-averaged phosphate level. The kinetic model of phosphate used in the analyses described here is a steady-state model. According to this model, weekly phosphate removal by hemodiafiltration or hemodialysis at equilibrium simply reflects the amount of phosphate absorbed during the week that has not been removed by residual kidney function.

## 9. Kinetic Modeling Comparison of Hemodialysis with Hemodiafiltration

In Table 1, we have modeled typical treatment variables for hemodialysis and hemodiafiltration. In the present example, we are assuming a replacement fluid infusion rate of 100 mL/min. This represents so-called “high-volume” hemodiafiltration for an average-sized patient undergoing a 4 h treatment, as 100 mL/min × 240 min = 24 L of substitution fluid. We further assume that one is using a dialyzer with an in vitro mass transfer area coefficient (K_0_A) for urea of 1200 mL/min. Given an assumed blood flow rate of 350 mL/min, after downsizing the in vitro urea K_0_A to an estimated in vivo value, the adjusted predicted in vivo dialyzer clearance for urea calculated by kinetic modeling (Solute Solver phosphate, version 3.03 [18]) is 239 mL/min (assuming a dialysate flow rate of 500 mL/min). In the case of hemodiafiltration, using the EuDial equations mentioned above to consider the added convective clearance, the predicted in vivo dialyzer urea clearance would be 255 mL/min, 7% higher. Assuming a 4 h treatment and a patient urea distribution volume of 35L, the expected urea reduction ratio (URR) values for hemodialysis and hemodiafiltration under these conditions would be 74.3 and 76.2%, respectively. Corresponding single-pool Kt/V urea values would be 1.59 and 1.68.

After adjusting the in vitro dialyzer K_0_A urea downward for phosphate (e.g., multiplying by 0.50 in this case), and making the appropriate in vivo/in vitro adjustment, and calculating clearance for phosphate based on plasma water instead of blood water, one finds that hemodiafiltration under these conditions, with a replacement fluid rate of 100 mL/min, is predicted to increase the in vivo dialyzer phosphate clearance of phosphate by 18%, from 152 mL/min with hemodialysis to 179 mL/min with hemodiafiltration (Table 1).

Table 2 shows four different scenarios under which hemodiafiltration can have different impacts on the predialysis serum phosphate level compared with hemodialysis, depending on the values for nondialysis-related factors. In scenarios 1, 2, and 3, we are assuming a residual kidney (water) urea clearance of 1.0 mL/min, corresponding to a residual kidney phosphate (water) clearance that is 20% higher, at 1.2 mL/min. In scenario 4, the impact of a reduced residual kidney clearance in a patient undergoing treatment with hemodiafiltration is explored.

mg, **Scenario1-hemodialysis**, 55, m, 3, 135, 1.0, **1.2**, 33, 35, 5, 350, 500, 240, 7, 75, 1200, 0.5, DZERNAME, **3.9**, **900**, 999, 0, 0, 0 mg, **Scenario1-hemodiafiltration**, 55, m, 3, 135, 1.0, **1.2**, 33, 35, 5, 350, 500, 240, 7, 75, 1200, 0.5, DZERNAME, **3.9**, **900**, 999, 0, 100, 0 mg, **Scenario2-hemodialysis**, 55, m, 3, 135, 1.0, 1.2, 33, 35, 5, 350, 500, 240, 7, 75, 1200, 0.5, DZERNAME, 3.9, 900, 999, 0, 0, 0 mg, **Scenario2-hemodiafiltration**, 55, m, 3, 135, 1.0, 1.2, 33, 35, 5, 350, 500, 240, 7, 75, 1200, 0.5, DZERNAME, **2.35**, **900**, 999, 0, 100, 0 mg, **Scenario3-hemodialysis,** 55, m, 3, 135, 1.0, 1.2, 33, 35, 5, 350, 500, 240, 7, 75, 1200, 0.5, DZERNAME, 3.9, 900, 999, 0, 0, 0 mg, **Scenario3-hemodiafiltration**, 55, m, 3, 135, 1.0, 1.2, 33, 35, 5, 350, 500, 240, 7, 75, 1200, 0.5, DZERNAME, **3.9**, **970**, 999, 0, 100, 0 mg, **Scenario4-hemodialysis**, 55, m, 3, 135, 1.0, **1.2**, 33, 35, 5, 350, 500, 240, 7, 75, 1200, 0.5, DZERNAME, 3.9, 900, 999, 0, 0, 0 mg, **Scenario4-hemodiafiltration**, 55, m, 3, 135, 0.33, **0.40**, 33, 35, 5, 350, 500, 240, 7, 75, 1200, 0.5, DZERNAME, 3.9, 900, 999, 0, 100, 0

**Scenario 1: Same phosphate ingestion, same PBED dose, and same residual kidney function with hemodialysis and hemodiafiltration.** In this example, modeling assumes that under both treatments, the patient is ingesting the same amount (900 mg/day) of phosphate and is being prescribed the same amount of binders, with the PBED being 3.9 g/day. Also, the level of residual kidney phosphate (water) clearance is the same: 1.2 mL/min. **Outcome:** Under such conditions, the model predicts, at steady state, a midweek predialysis serum phosphate value of 4.35 mg/dL with hemodialysis vs. 3.85 mg/dL with hemodiafiltration, a 0.50 mg/dL lower value with hemodiafiltration.

**Scenario 2: Same phosphate ingestion and same residual kidney function with hemodialysis and hemodiafiltration, but lower PBED with hemodiafiltration.** Here, the dialytic treatment and patient factors remain the same as in Scenario 1. Dietary phosphate ingestion remains at 900 mg/day with both treatments, and residual kidney function is also the same. However, the PBED in the hemodiafiltration case is reduced. **Outcome:** Modeling predicts that a reduction in PBED from 3.9 to 2.4 g/day (−1.5 g/day) would result in the predialysis serum phosphate rising from 3.85 mg/dL back up to 4.35 mg/dL with hemodiafiltration, nullifying its lowering of predialysis serum phosphate.

**Scenario 3: Same PBED and same residual kidney function with hemodialysis and hemodiafiltration, with higher phosphate ingestion in hemodiafiltration.** Here, the dialytic treatment and patient factors remain the same as in Scenario 1. PBED and residual kidney function are the same with both treatments. However, during treatment with hemodiafiltration, the patient is modeled to consume a slightly higher amount of phosphate compared with when he or she was treated with hemodialysis (e.g., 970 vs. 900 mg/day). **Outcome:** Modeling predicts that with this slightly higher phosphate ingestion, the predialysis serum phosphate with hemodiafiltration will no longer be different from that with hemodialysis.

**Scenario 4: Same phosphate ingestion and same PBED with hemodialysis and hemodiafiltration but lower residual kidney function during hemodiafiltration.** Here, the dialytic treatment and patient factors remain unchanged. Dietary phosphate ingestion is assumed to be 900 mg/day in both instances, and the PBED is also the same (3.9 g/day). However, residual kidney phosphate (water) clearance is slightly lower with hemodiafiltration (0.40 mL/min vs. 1.2 mL/min during hemodialysis). **Outcome:** Similar modeled predialysis serum phosphate levels in hemodiafiltration and hemodialysis.

## 10. Influence of Residual Kidney Function and Baseline Predialysis Serum Phosphate on Hemodiafiltration vs. Hemodialysis Comparisons

Kinetic modeling suggests that in anuric patients, the advantage of hemodiafiltration vs. hemodialysis on predialysis serum phosphate levels will be magnified to some extent. For example, in Scenario 1 above, if we reduce kidney function to zero and also lower phosphate ingestion from 900 to 794 mg/day in order to maintain a comparable level of predialysis serum phosphate with hemodialysis (4.35 mg/dL), with hemodiafiltration, our modeling predicts that the predialysis serum phosphate level would be 3.74 mg/dL, i.e., 0.61 mg/dL lower instead of 0.50 mg/dL lower if residual kidney phosphate (water) clearance were 1.2 mL/min.

The baseline level of predialysis serum phosphate with hemodialysis is also a factor. For example, if one increases dietary phosphate ingestion to 1200 mg/day and assumes anuria with a PBED of 3.9 g/day, the modeled predialysis serum phosphate level in hemodialysis in this modified Scenario 1 rises to 8.71 mg/dL. With hemodiafiltration, the predicted value is 8.01 mg/dL, which is now 0.7 mg/dL lower than with hemodialysis.

## 11. Concordance of Kinetic Modeling Results with Experimental Measures of Phosphate Removal Comparing Hemodiafiltration with Hemodialysis

In a prior publication, the Solute Solver phosphate kinetic model was used to analyze five studies reported by others comparing hemodiafiltration with hemodialysis, which included measurements of phosphate removal in spent dialysate [49]. In each of these five studies, the predialysis serum phosphate values prior to hemodiafiltration and hemodialysis were similar by design. These were acute studies, and they were not intended to study the impact of hemodiafiltration vs. hemodialysis on the predialysis serum phosphate level during extended use. The hemodiafiltration replacement fluid infusion rate ranged from 50 to 100 mL/min. The average modeled amounts of phosphate removed by hemodiafiltration or hemodialysis ranged from 5 to 13% higher with hemodiafiltration compared with hemodialysis. The predicted phosphate removal advantage using the Solute Solver phosphate kinetic model was similar to the reported measured values [49].

## 12. Major Randomized Comparisons of Hemodiafiltration and Hemodialysis

There have been five relatively large randomized controlled clinical trials comparing hemodiafiltration with hemodialysis [50,51,52,53,54]. The average baseline and follow-up predialysis serum phosphate values in each trial are shown in Table 3. As can be seen, there was little difference in follow-up serum phosphate values between the hemodiafiltration and hemodialysis groups. One exception where hemodiafiltration was shown to have a benefit was a post hoc analysis of the CONTRAST trial reported by Penne et al. [55]. Here, the proportion of patients reaching phosphate treatment targets increased from 64% to 74% in hemodiafiltration patients, while the percentage of hemodialysis patients reaching the target was unchanged at 66% (*p* = 0.04).

## 13. Selected Crossover Comparisons of Hemodiafiltration with Hemodialysis in Studies That Reported Serum Phosphate Levels

A number of authors have studied the effects of hemodiafiltration on predialysis serum phosphate using crossover designs, which may be useful as they limit between-patient variance. Vega-Vega and colleagues (Table 4) compared predialysis serum phosphate with high flux hemodialysis, online postdilution hemodiafiltration with a replacement fluid rate of 25 L over a 4 h treatment, and so-called expanded dialysis (HDx) using a medium cutoff membrane [56]. They used a balanced crossover design: they divided the patients into three groups, each starting with a different therapy. With hemodialysis, mean predialysis serum phosphate increased slightly, while after 4 weeks of treatment with hemodiafiltration (or, to a lesser extent, with HDx), the predialysis serum phosphate decreased by approximately 1.0 mg/dL. Residual kidney function was not measured, but the average dialysis vintage was more than 4 years. When their data are analyzed using the Solute-Solver phosphate kinetic model, assuming no change in residual kidney function or phosphate binder dose (the amount of which was not reported) across the three treatments, modeling predicts a decrease in predialysis serum phosphate during the hemodiafiltration period of about 1.0 mg/dL, very similar to the measured value.

Assuming an average patient urea volume of 34 L, a phosphate ingestion of 910 mg/day, a PBED of 5.0 g/day, no residual kidney function, and dialyzer efficacy, blood flow, dialysate flow, and session length as reported in their paper [56], and an hemodiafiltration replacement fluid rate of 104 mL/min, one can use the following input file for the Phosphate Solver modeling program:
mg, **vega-hemodialysis**, 36, m, 3, 135, 0, 0, 33, 34, 7, 383, 500, 240, 7, 64, 1200, 0.5, DZER, 5.0, 910, 999, 0, 0, 0 mg, **vega-hemodiafiltration**, 36, m, 3, 135, 0, 0, 33, 34, 7, 383, 800, 240, 7, 64, 1400, 0.5, DZER, 5.0, 910, 999, 0, 104, 0

With such an input file, the Solute Solver phosphate kinetic modeling program predicts a 3.9 mg/dL predialysis serum phosphate with hemodiafiltration at steady-state vs. 4.9 mg/dL with hemodialysis, close to what was measured.

Why the difference of 1.0 mg/dL in predialysis serum phosphate between hemodiafiltration and hemodialysis in the Vega-Vega study, while in Table 2, the modeled difference was only 0.5 mg/dL? In the patients treated by Vega-Vega et al. [56], the dialyzers used for hemodiafiltration had a higher efficiency than those used for hemodialysis. Also, the total dialysate + hemodiafiltration fluid flow rate was increased during the hemodiafiltration period from 500 to 800 mL/min, increasing phosphate clearance.

In another crossover trial, by Pedrini and colleagues [57], 62 patients were treated with hemodiafiltration and hemodialysis. Again, the treatments were sequenced in a balanced fashion. Most of the patients were treated with postdilution hemodiafiltration, though three patients were treated using predilution, and 13 patients received mixed pre/postdilution hemodiafiltration. The replacement fluid rate was 58 mL/min. The duration of each treatment was 6 months. At the end of the hemodiafiltration treatment, the average predialysis serum phosphate was 4.6 mg/dL, compared with 5.0 mg/dL at the end of the hemodialysis period, a difference that was highly significant. Dietary phosphate ingestion was not assessed, and the phosphate binder dose was not reported.

A third crossover trial done in 2017 by Smith et al. [58] studied 100 patients, again using a balanced order of treatments. Eighty-six patients completed the trial. Each patient received 8 weeks of hemodialysis and 8 weeks of postdilution hemodiafiltration. The average session length was 250 min, and the replacement fluid rate averaged 20 L per session. During the hemodiafiltration periods, the patients had an increased risk of intradialytic hypotension (an unexpected finding), and there was also an increased risk of circuit clotting. In this particular trial, there was no improvement in predialysis serum phosphate with hemodiafiltration compared with hemodialysis. Dietary phosphate ingestion was not assessed, and the phosphate binder dose was not reported.

A report by Abdelsalam [59] surveyed patients treated in Saudi Arabia. It was partly an observational and partly an interventional study. In 900 patients treated by hemodialysis, the mean predialysis serum phosphate levels averaged 4.8 mg/dL. In 215 patients selected for hemodiafiltration, because they “did not achieve adequacy targets” or because “adequate phosphorus control could not be achieved with standard procedures after 3 months,” baseline predialysis serum phosphate was 5.5 mg/dL, and this was reduced to 5.16 mg/dL after 90 days of treatment with hemodiafiltration, using a slightly higher blood flow rate and larger dialyzer.

Another crossover trial by Movilli et al. [60] reported changes in serum phosphate as well as changes in phosphate binder dose in 30 patients out of 225 who were changed to hemodiafiltration. The controls were treated with low-flux hemodialysis. The replacement fluid rate was set at 35% of the blood flow rate. As shown in Table 4, in the patients who changed to hemodiafiltration, the predialysis serum phosphate decreased from 5.1 to 4.0 mg/dL, whereas it was unchanged in the concurrent controls who remained on low-flux hemodialysis. Phosphate binder doses were largely unchanged in both groups.

## 14. Selected Observational Data Comparing Phosphate Control with Hemodiafiltration vs. Hemodialysis

Given the potential impact of differences in dietary phosphate ingestion, phosphate binder prescription, and level of residual kidney phosphate clearance, one must evaluate observational data comparing predialysis serum phosphate levels between patients treated with hemodiafiltration or hemodialysis very carefully, especially when these other factors have not been measured or reported. Still, so-called “real-world” data have their utility. Two of the largest observational studies that reported phosphate control with hemodiafiltration are the Pan Thames audit, reported by Davenport et al. [61], and data from DOPPS, reported by Locatelli and colleagues [62]. As shown in Table 5, in the Pan Thames audit, the predialysis serum phosphate level was on average about 0.33 mg/dL lower in patients being treated with hemodiafiltration compared with those treated with hemodialysis, and this was found despite a slightly shorter treatment time in the hemodiafiltration patients [61]. The replacement fluid volume was reported to be in the 15–20 L/treatment range. As in many other comparisons, no data were reported regarding dietary phosphate ingestion or phosphate binder dose. The large observational comparison published by Locatelli and colleagues using the DOPPS database (62) was useful in that subjects undergoing hemodiafiltration were grouped according to the per treatment volume of replacement fluid used: 4–15, 15–20, and >20 L. With regard to phosphate control, the average predialysis value in patients treated with hemodialysis was 5.0 mg/dL, while it was 4.8–4.9 mg/dL in patients treated with hemodiafiltration, with the lower mean value of 4.8 mg/dL found in patients treated with hemodiafiltration with replacement fluid volume > 20 L per treatment. The potential statistical significance of the small differences in predialysis serum phosphate between the “high-dose” hemodiafiltration and hemodialysis groups was not reported. A substantial proportion of patients had some residual kidney function (30–42% on average in the various subgroups).

**Figure 2:**
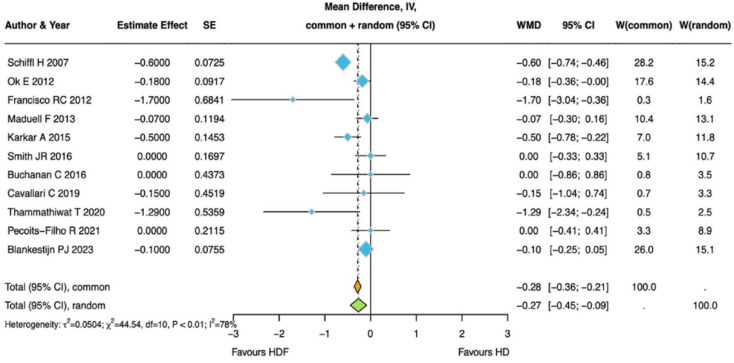
Predialysis serum phosphate levels in 11 randomized controlled trials comparing hemodiafiltration with hemodialysis. Reproduced with permission from Battaglia et al. *Nephrol Dial Transplant.* 2025 Aug 1;40(8):1590–1614 [63].

There are a number of additional small, randomized trials and observational cohort studies that have reported phosphate data in patients treated with hemodiafiltration or hemodialysis. Most of them involved fewer than 200 patients. An exhaustive review is beyond the scope of the present analysis. A formal systematic review of the effects of hemodiafiltration vs. hemodialysis on serum phosphate was included in a report by Battaglia as part of a European Renal Association (ERA) consensus statement on hemodiafiltration [63]. One of their meta-analyses comparing phosphate data in 11 randomized trials is shown in Figure 2.

One recent prospective cross-sectional observational study that does warrant mention was done by Hegbrant et al. [39]. It was done in four clinics in Spain, two of which treated all of their patients with hemodialysis, and two which treated almost all patients with hemodiafiltration. This study is unique in that the main nondialysis factors affecting predialysis serum phosphate, namely, phosphate intake, PBED, and residual kidney phosphate clearance, were all measured and included in a kinetic modeling analysis. The patient demographics in the four clinics were broadly similar, and the proportions of patients with residual kidney function were comparable in the hemodialysis and hemodiafiltration clinics. The predialysis serum phosphate levels in the hemodiafiltration clinics were not lower than in the hemodialysis clinics (Table 5). However, there was a trend for a reduction in phosphate binders in the hemodiafiltration clinics when expressed as PBED, similar to findings reported by Oates et al. [64]. Kinetic modeling suggested that dietary phosphate intakes in the hemodialysis and hemodiafiltration clinics would be similar, but dietary assessment by a food frequency questionnaire suggested that the phosphate ingestion in the hemodiafiltration clinic patients was higher than in the clinic patients being treated with hemodialysis. The study showed the importance of residual kidney function, in that among all patients, those with substantial residual kidney function were being prescribed substantially lower amounts of phosphate binders, and yet the mean predialysis serum phosphate in this subgroup was not increased.

## 15. Dialysate Plus Replacement Fluid Flow Rate, Blood Flow Rate, and Dialyzer Efficiency in Comparisons of Hemodiafiltration with Hemodialysis

One of the problems with analyzing data from, especially, non-randomized trials comparing hemodiafiltration with hemodialysis is that quite often, when treatment is changed from hemodialysis to hemodiafiltration, a larger dialyzer is used, which may not only have a higher urea clearance, but may also have been optimized to increase phosphate removal. In many studies, blood and dialysate flow rates are often increased in the hemodiafiltration group relative to hemodialysis. A recommendation has been made to attempt delivery of a convective replacement fluid rate of at least 23 L per session when performing hemodiafiltration, which translates to about 100 mL/min for a session with a duration of 4 h [63]. Does the replacement fluid rate impact the reduction in serum phosphate? One can model this in the data reported by Vega-Vega et al. [56], and substitute 104 mL/min with 50 mL/min as the replacement fluid rate. Here is the input file that one can use for Phosphate Solver [19]:
mg, **vega-hemodialysis**, 36, m, 3, 135, 0, 0, 33, 34, 7, 383, 500, 240, 7, 64, 1200, 0.5, DZER, 5.0, 909, 999, 0, **0**, 0 mg, **vega-hemodiafiltration-104**, 36, m, 3, 135, 0, 0, 33, 34, 7, 383, **800**, 240, 7, 64, 1400, 0.5, DZER, 5.0, 909, 999, 0, **104**, 0 mg, **vega-hemodiafiltration-50**, 36, m, 3, 135, 0, 0, 33, 34, 7, 383, **800**, 240, 7, 64, 1400, 0.5, DZER, 5.0, 909, 999, 0, **50**, 0 mg, **vega-hemodiafiltration-50**, 36, m, 3, 135, 0, 0, 33, 34, 7, 383, **500**, 240, 7, 64, 1200, 0.5, DZER, 5.0, 909, 999, 0, **104**, 0


The phosphate kinetic model predicts that, if the replacement fluid rate had been 50 instead of 104 (approximately 12 L instead of 25 per treatment; row 3 above), that the pretreatment serum phosphate level would still be lowered by hemodiafiltration relative to hemodialysis, but from 4.9 mg/dL to 4.15 instead of from 4.9 to 3.9, a decrease of 0.75 mg/dL instead of 1.0. What about lowering the dialysate + hemodiafiltration flow rate? In the Vega-Vega study, the model predicts that, if the same dialyzer had been used, and if the total dialysate plus hemodiafiltration fluid flow rate had been set at 500 mL/min instead of 800 mL/min, that the predialysis serum phosphate would have decreased to 4.13 mg/dL instead of to 3.9. Thus, modeling suggests that there are small benefits in terms of serum phosphate reduction both for an increased dialysate flow rate and for an increased replacement fluid rate. This model prediction is somewhat at odds with a multivariable analysis of treatment factors affecting serum phosphate by Jaques et al. [65] in a small prospective observational study in which phosphate removal was measured directly. Their analysis suggested that replacement volume was not related to the lowering of the predialysis serum phosphate level.

Recently, attention has focused on the potential economic advantages and lack of substantial clearance disadvantages of keeping the dialysate plus hemodiafiltration fluid flow rate unchanged when switching from hemodialysis to hemodiafiltration [66,67], and also tying the replacement fluid infusion rate to the blood flow rate. The described kinetic model for phosphate, which relies on the EuDial convective clearance equations, can be used to predict the effect of changes in therapy parameters, such as dialysate and replacement fluid flow rates, on predialysis serum phosphate. However, there are no published data that systematically attempt to validate the EuDial equation predictions with regard to phosphate clearance for hemodiafiltration. This especially holds true for predilution and mixed-dilution hemodiafiltration. The Solute Solver phosphate kinetic modeling data suggest that with predilution hemodiafiltration, one should expect no reduction in predialysis serum phosphate, while with mixed hemodiafiltration (50% pre and 50% post), the achieved reduction in predialysis serum phosphate would be half of what can be expected compared with the same total replacement volume given in postdilution mode [49].

Limitations: All models depend on input assumptions. With the phosphate kinetic model used in the analyses reported here, assumptions include in vivo dialyzer phosphate clearance values based on the urea in vitro mass transfer area coefficient (K_0_A), subjected to a number of in vivo/in vitro adjustments, with convective clearance added based on the EuDial equations. With regard to phosphate absorption, an average phosphate absorption percentage of 67% is assumed, and we know this can vary depending on the type of diet eaten and the percentage of phosphate ingested from food additives. More importantly, the model assumes a steady-state system, whereas the predialysis serum phosphate can rapidly change if there are changes in phosphate ingestion due to illness or binge eating, or simply due to ordinary day-to-day variability. The reliability of food frequency questionnaires to characterize diet is not optimal [68], although at least one group has found it to achieve reproducible results [69]. The kinetic model of phosphate described here assumes that each gram of phosphate binder (as PBED) binds to 45 mg of phosphate, and this amount may vary somewhat among binders. Prescribed PBED is different from ingested PBED, and compliance in taking any medication complicates the measurement of drug efficacy. Residual kidney phosphate clearance is not routinely measured in dialysis patients, and inferring phosphate clearance by adjusting the measured residual kidney urea clearance may work in terms of average values, but the ratio of residual kidney phosphate to urea clearance can vary substantially among patients, and perhaps even in the same patient, when urine is collected under different conditions.

While the phosphate kinetic model used has been validated in groups or subgroups of patients in terms of predicting average values of intradialysis and early postdialysis serum phosphate levels [16], the amount of phosphate removed [17,49], and dietary phosphate ingestion [39,70], it has not been tested in terms its ability to predict changes in serum phosphate in individual patients in response to a change in dialysis therapy, PBED, phosphate intake, or residual kidney function. The present phosphate model cannot be used to model long and frequent nocturnal dialysis and similar therapies, as it fails to accurately model intradialysis levels of serum phosphate for sessions longer than 5 h.

## 16. Summary

Kinetic modeling predicts that (high-volume) hemodiafiltration achieves greater phosphate clearance (by about 15–20%) than conventional hemodialysis; however, this does not always translate into lower predialysis serum phosphate in hemodiafiltration-treated patients. Predialysis phosphate values are affected not only by the amount of extracorporeal removal, but also by the amount of dietary phosphate ingestion (and percent absorption), phosphate-binder dose, and residual kidney phosphate clearance. When these three nondialytic factors are held constant, then kinetic modeling predicts that (high-volume) hemodiafiltration will result in an expected reduction of ~0.5 mg/dL in predialysis serum phosphate relative to hemodialysis (Table 2, scenario 1). This advantage can be completely offset by relatively modest changes in any of three other determinants affecting serum phosphate. For example, an ~8% increase in phosphate ingestion, a reduction in the phosphate binder equivalent dose (PBED) of 1.5 g/day, or a decline in residual kidney phosphate clearance of 0.8 mL/min in patients treated with hemodiafiltration will result in no diminution of predialysis serum phosphate levels relative to hemodialysis (Table 2, scenarios 2, 3, and 4, respectively). Changes in these other three determinants (either singly or in combination) can explain findings in studies that found no differences in predialysis serum phosphate between hemodiafiltration and hemodialysis.

## Figures and Tables

**Table 1:** Kinetic modeling predictions illustrating differences in urea and phosphate clearances between hemodialysis and hemodiafiltration.

Modeled Value	Hemodialysis	Hemodiafiltration
Qb (mL/min)	350	350
Qd (mL/min)	500	500 *
Hemodiafiltration replacement fluid rate (mL/min)	0	100
In vitro dialyzer K_0_A urea (mL/min)	1200	1200
Session length (min)	240	240
Kdurea (mL/min)	239	255 (+7% hemodiafiltration vs. hemodialysis)
Kdphos (mL/min)	152	179 (+18% hemodiafiltration vs. hemodialysis)
Weekly fluid removal (L)	7.0	7.0
Urea distribution volume (L)	35	35
URR (%, urea reduction percentage)	74.3	76.2
Kt/V-urea (single-pool)	1.59	1.68

Qb = blood flow rate; * Qd = total dialysate flow rate, including the 100 mL/min of hemodiafiltration replacement fluid; K_0_A = in vitro mass transfer area coefficient of dialyzer; Kdurea = in vivo-modeled dialyzer urea (blood water) clearance; Kdphos = in vivo-modeled dialyzer phosphate (plasma water) clearance.

**Table 2:** Four different scenarios showing the impact of slight differences in PBED, dietary phosphate ingestion or residual kidney function with hemodiafiltration on the predialysis serum phosphate level.

**Scenario 1: Same phosphate ingestion with hemodialysis and hemodiafiltration (900 mg/day); same PBED (3.9 g/day); same levels of residual kidney function (1.2 mL/min residual kidney phosphate (water) clearance.**		
Phosphate ingestion (mg/day)	900	900
PBED (g/day)	3.9	3.9
Residual kidney phosphate (water) clearance (mL/min)	1.20	1.20
Phosphate reduction ratio (%)	51.2	53.0
Predicted predialysis serum phosphate (mg/dL)	4.35	3.85 (−0. 50 mg/dL)
*Outcome:* With hemodiafiltration, lower predialysis serum phosphate by 0.50 mg/dL relative to hemodialysis.		
**Scenario 2: Same phosphate ingestion with hemodialysis and hemodiafiltration and same levels of residual kidney function, but PBED with hemodiafiltration lower (2.4 g/day instead of 3.9).**		
Phosphate ingestion (mg/day)	900	900
PBED (g/day)	3.9	2.4 (−1.5 g/day)
Residual kidney phosphate (water) clearance (mL/min)	1.20	1.20
Phosphate reduction ratio (%)	51.2	54.6
Predicted predialysis serum phosphate (mg/dL)	4.35	4.35
*Outcome:* Predialysis serum phosphate similar with hemodiafiltration and hemodialysis. Dose savings of 1.5 g/day PBED with hemodiafiltration.		
**Scenario 3: Same PBED (3.9 g/day) with hemodiafiltration and hemodialysis and same residual kidney function (1.2 mL/min residual kidney phosphate (water) clearance), but phosphate ingestion 970 mg/day with hemodiafiltration vs. 900 mg/day with hemodialysis.**		
Phosphate ingestion (mg/day)	900	970
PBED (g/day)	3.9	3.9
Phosphate reduction ratio (%)	52.1	54.3
Predicted predialysis serum phosphate (mg/dL)	4.35	4.35
*Outcome:* Predialysis serum phosphate similar with hemodiafiltration and hemodialysis.		
**Scenario 4: Same phosphate ingestion with hemodiafiltration and hemodialysis (900 mg/day), and same PBED (3.9 g/day), but residual kidney phosphate (water) clearance is lower during hemodiafiltration (0.40 mL/min vs. 1.2 mL/min with hemodialysis).**		
Phosphate ingestion (mg/day)	900	900
PBED (g/day)	3.9	3.9
Residual kidney phosphate (water) clearance (mL/min)	1.20	0.40
Phosphate reduction ratio (%)	52.1	54.4
Predicted predialysis serum phosphate (mg/dL)	4.35	4.35
*Outcome:* Predialysis serum phosphate similar with hemodiafiltration and hemodialysis.		

PBED, phosphate binder equivalent dose. These are the phosphate Solver version 3.03 [18] inputs for the four scenarios above. For variable name identification, see the program specification. UNITS (mg or mmol), PTID, AGE, SEX, LABDAYOFWK, SCHEDULE, KRU, KRPHOS, HCT, UREAVOL, UREAGEN, QB, QD, TD, WEEKLYFLUIDLOSS, POSTWEIGHTKG, K_0_A_UREA, KOA_PU_RATIO, DZERNAME, EBD, DIP, PREPHOS, HDFPREDIL, HDFPOSTDIL, and QDPLUS.

**Table 3:** Hemodiafiltration vs. hemodialysis effects on predialysis serum phosphate in five selected major randomized trials.

Study (First Author, Study Nickname), Ref.	N of Cases Hemodiafiltration/Hemodialysis	Baseline Predialysis Serum Phosphate (Hemodiafiltration) mg/dL	Follow-Up Predialysis Serum Phosphate (Hemodiafiltration) mg/dL	Baseline Predialysis Serum Phosphate (Hemodialysis) mg/dL	Follow-Up Predialysis Serum Phosphate (Hemodialysis) mg/dL
Grooteman (CONTRAST) [50]	358/356	5.12	4.80	5.05	4.95
Ok (TURKISH) [52]	391/391	5.13 L/4.72 H *	4.78 L/4.54 H *	4.88	4.72
Maduell (ESHOL) [51]	456/450	4.73	4.49	4.58	4.69
Morena (FRENCHIE) [53]	190/191	4.50	4.00	4.50	4.34
Blankestijn (CONVINCE) [54]	683/677	4.90	4.8	4.95	4.9

The predialysis serum phosphate values are the reported mean values for each randomized treatment arm. The hemodialysis comparator groups were high-flux dialysis in all studies shown except the CONTRAST trial, where the comparator group was low-flux. * Low/high (L,H)-volume hemodiafiltration. In the Turkish trial by Ok et al. [52], the hemodiafiltration group was divided into low- and high-volume subgroups. The Maduell et al. [51] follow-up phosphate values shown are those reported at 6 months of follow-up. Using a variety of statistical tests, follow-up phosphate values were reported as significantly different with hemodiafiltration in the studies by Ok et al. [52] (with high volume hemodiafiltration) and in the studies by Maduell et al. [51] and Morena et al. [53] Follow-up values were not reported as significantly different in the primary trial result reports by Grooteman et al. [50] or Blankestijn et al. [54].

**Table 4:** Hemodiafiltration vs. hemodialysis effects on predialysis serum phosphate in several major crossover trials.

Study (First Author, Ref.)	N. of Patients Completing Trial	Baseline Predialysis Serum Phosphate (Hemodiafiltration) mg/dL	Follow-Up Predialysis Serum Phosphate (Hemodiafiltration) mg/dL	Baseline Predialysis Serum Phosphate (Hemodialysis) mg/dL	Follow-Up Predialysis Serum Phosphate (Hemodialysis) mg/dL
Vega-Vega [56]	22 (crossover)	4.9	3.9	4.9	5.0
Pedrini * [57]	62 (crossover)	NA	4.6	NA	5.0
Smith [58]	86 (crossover)	NA	5.0	NA	5.0
Abdelsalam * [59]	215 crossover)	5.5	5.16	NA	NA
Movilli * [60]	30 (crossover)	5.3	4.0	5.0	5.2

* Low-flux hemodialysis was the comparator treatment. Using a variety of statistical tests, follow-up phosphate values were reported as significantly different with hemodiafiltration in the study by Pedrini et al. [57]. The binder dose of sevelamer was reduced in the hemodiafiltration period in the study by Pedrini et al. [57]. The changes in predialysis serum phosphate in the crossover trial by Vega-Vega were reported as significant. NA, not available in the published report.

**Table 5:** Selected observational comparisons of hemodiafiltration with hemodialysis.

Study (Author, Ref.)	N. of Cases Hemodiafiltration	N. of Cases Hemodialysis	Predialysis Serum Phosphate (Hemodiafiltration) mg/dL	Predialysis Serum Phosphate (Hemodialysis) mg/dL	Comments
Davenport (Pan Thames) [61]	851	4515	4.40	4.73	*p* < 0.001
Locatelli (DOPPS) [62]	2012	6555	4.9 4.8 when hemodiafiltration volume ≥ 15 L	5.0	*p*-value not reported
Hegbrant [39]	56	59	4.60	4.37	*p* NS

## Data Availability

No new data were created or analyzed in this study. Data sharing is not applicable to this article. The kinetic phosphate model used in the analyses is available at http://www.ureakinetics.org (version 3.03) accessed on 23 March 2026. Username = solute and password = solver.
